# Risk‐stratified introduction of precautionary allergen‐labeled foods in children with peanut and tree nut allergies

**DOI:** 10.1111/pai.70306

**Published:** 2026-02-27

**Authors:** François Graham, Mikaël Frossard, Natalia Gasilova, Fabienne Maréchal, Philippe A. Eigenmann

**Affiliations:** ^1^ Pediatric Allergy Unit, Department of Child and Adolescent University Hospitals of Geneva Geneva Switzerland; ^2^ Division of Allergy and Clinical Immunology, Department of Medicine Centre Hospitalier de l'Université de Montréal Montreal Quebec Canada; ^3^ Department of Pediatric Allergy and Immunology Centre Hospitalier Universitaire Sainte‐Justine Montreal Quebec Canada; ^4^ Laboratoire d'Électrochimie Physique et Analytique, EPFL Valais Wallis École Polytechnique Fédérale de Lausanne Sion Switzerland; ^5^ Mass Spectrometry and Elemental Analysis Platform, ISIC‐GE‐VS, EPFL Valais Wallis École Polytechnique Fédérale de Lausanne Sion Switzerland; ^6^ Clinical Research Plateform, Department of Child and Adolescent University Hospitals of Geneva Geneva Switzerland

**Keywords:** food allergy, nut allergy, oral food challenge, peanut allergy, pediatrics, precautionary allergen labeling, traces

To the Editor,

For patients living with food allergies, a major source of stress is the fear of accidentally reacting to small amounts of allergens in processed foods, including foods with precautionary allergen labeling (PAL) such as “may contain”.^1^, ^2^ PAL is a label warning on pre‐packaged foods indicating the potential unintentional presence of a priority allergen, but it remains largely unregulated in most countries, leaving its use to manufacturers' discretion. Various studies have investigated the presence of allergen contamination in foods with PAL, generally showing an absence or only low‐grade contamination.^1^, ^3^ The majority of patients who strictly avoid these foods report that this avoidance diet causes significant disruptions in daily life, particularly in social settings and restaurants. Unsurprisingly, up to 40% of food‐allergic patients eventually introduce these foods,^4^ with a large proportion not experiencing any reaction upon consumption.^5^ Nevertheless, some patients with more severe allergy phenotypes could potentially experience anaphylaxis after exposure to these foods, and personalized recommendations are sometimes adopted by treating physicians.^6^ Individualized management approaches towards PAL have recently been proposed based on the result of a single‐dose oral food challenge (OFC) as a decision point.^7^, ^8^, ^9^ In this context, a single‐dose OFC could be offered with a quantity corresponding to a defined threshold dose to stratify patients into high‐risk versus low‐risk of reacting to foods with PAL. We hypothesized that high‐threshold reactors would be at lower risk of reacting to foods with PAL and could safely introduce these foods. The objective of this study was to evaluate the feasibility, safety, and quality‐of‐life (QoL) impact of this strategy in real‐life practice.

Subjects aged 2–18 years of age with a confirmed peanut and/or tree nut allergy were recruited at the University Hospitals of Geneva and CHU Sainte‐Justine. All patients had a positive single‐dose single‐blind or double‐blind‐placebo‐controlled OFC to 30 mg of protein of their culprit peanut or tree nut. Only patients with a mild reaction were included in the study, with exclusion of patients with moderate or severe reactions at this dose (see Appendix S1 for the complete inclusion and exclusion criteria list and the severity grading scale used). When multiple peanut/tree nut allergies were present, the culprit allergen for eligibility confirmation was determined based on clinical judgment. The study was approved by the ethics committees of the University Hospitals of Geneva and CHU Sainte‐Justine. Written informed consent was obtained from parents or legal guardians of all participating children, with age‐appropriate assent obtained from the participants when applicable, in accordance with approval from the local ethics committees. The planned sample size was 35, but the study ended after 13 participants due to recruitment difficulties. The study is therefore underpowered, and all results should be interpreted as exploratory and hypothesis‐generating.

Included subjects underwent an open OFC with three processed foods labeled with “may contain traces”, following established safety and accuracy guidelines for food challenges.^10^ The following foods were given successively in a single dose equivalent to a regular serving size: cookies (maximum 30 g), chocolate (maximum 30 g), breakfast cereals (maximum 50 g). Each food was administered with a 1‐h observation period between doses and at the end of the challenge.

If a participant did not react to any of the foods or had only mild localized symptoms such as oral itching, they were instructed to discontinue avoidance of “may contain” foods and to integrate them regularly into the diet, with rescue medication and use instructions provided according to guidelines. Participants experiencing moderate or severe reactions were withdrawn and advised to avoid foods with PAL.

For the following 3 months, patients who successfully passed the OFCs were invited to eat on a regular basis any foods labeled as “may contain” tree nuts and/or peanuts and to record their consumption in a diary. Any adverse reactions were recorded and reported to the study team within 1 week, with an optional clinical visit scheduled if necessary. The brand name of the food, and if available, the batch number, was recorded. Patients were re‐evaluated after 3 months, when the parents/patients reported on the brand, frequency, and amount of “may contain” foods eaten.

Randomly selected samples of foods with PAL (3/13 cookies, 8/13 cereals, and 8/13 chocolates) used during OFCs were analyzed at the Laboratory of Physical and Analytical Electrochemistry of the Swiss Federal Technology Institute of Lausanne to assess for allergen residues. Allergen content was analyzed by proteomics approach using liquid chromatography coupled to high resolution tandem mass spectrometry (LC–MS/MS) (see Appendix S1).

Age‐appropriate Food Allergy Quality of Life Questionnaires (FAQLQ child form (CF) for children 7–12 years, parent form (PF) for parents with children 0–12 years, and teen form (TF) for children 13–17 years) were completed by the parents/patient at the beginning of the study and after 3 months. Given the small cohort, analyses were descriptive and hypothesis generating. Paired comparisons were performed using Wilcoxon rank‐sum tests.

Thirteen patients were included in the study with a median age of 11.5 years. Baseline characteristics are presented in Table 1. Three of 13 patients (23%) reacted during the OFCs with three commonly consumed foods labeled with PAL: chocolate, cookies, and breakfast cereals. The three patients who experienced reactions all had hazelnut allergies and exhibited mild oral pruritus following consumption of two different chocolate samples:

- One patient consumed a chocolate sample with no detectable hazelnut traces.
- The remaining two patients ingested a chocolate sample containing 112 ppm of hazelnut protein (equivalent to 3.36 mg of hazelnut protein per 30 g of chocolate).

**TABLE 1:** Clinical characteristics of participants.

Demographics	
Age in years, median (range)	11.5 (6.25–14.75)
Sex: female	5/13
History of past or current atopic diseases	
Atopic dermatitis	7/13
Allergic rhino‐conjunctivitis	10/13
Asthma	9/13
Food allergies other than nuts	7/13
Peanut/Nut allergies	
Almond allergy	4/13
Brazil nut allergy	2/13
Cashew allergy	8/13
Pine nut allergy	1/13
Pecan nut allergy	1/13
Macadamia nut allergy	3/13
Pistachio allergy	6/13
Peanut allergy	11/13
Hazelnut allergy	8/13
Walnut allergy	6/13
Sesame allergy	3/13
Number of peanut/nut allergies (median/range)	4 (1–11)
Specific IgE (median/range)	
Almond	0.49 (0–0.98)
Brasil nut	0.47 (0.‐0.94)
Cashew	2.55 (0.6–8.93)
Pine nut	0 (0–0)
Pecan nut	NA
Macadamia	0.59 (0.59–0.59)
Pistachio	1.62 (0.55–12.30)
Peanut	60.75 (3.61–100)
Ara h 2	35.35 (1.94–100)
Hazelnut	5.81 (0.15–28.70)
Cor a 9	0.67 (0.29–1.5)
Cor a 14	5.92 (2.05–24.2)
Walnut	11.62 (1.16–22.4)
Sesame	0.87 (0.16–1.57)

In addition to the chocolates, allergen traces were also evaluated in other food samples:

- Cookies: A batch containing 12.1 ppm of peanut protein (0.363 mg per 30 g) was provided to three peanut‐allergic patients. None experienced a reaction.
- Cereals: Two cereal batches were tested for peanut contamination, but no peanut protein was detected. Eight peanut‐allergic patients consumed these cereals, with none experiencing a reaction.
- Chocolates: Four batches were tested for hazelnut contamination and provided to eight hazelnut‐allergic patients. Three batches contained detectable levels of hazelnut protein (ranging from 23.5 to 126.2 ppm). Of the eight patients, three reacted (as mentioned above), while five did not. Notably, two of the non‐reacting patients had a known hazelnut allergy.

During the three‐month follow‐up of patients regularly consuming foods with PAL, all patients incorporated chocolates and cookies with PAL into their diets, while 11 out of 13 patients consumed cereals with PAL. Additionally, 10 out of 13 participants introduced other food categories with PAL, including appetizer biscuits, ice cream, various pastries and viennoiseries, ice cream, ravioli, chips, cereal bars, fruit paste, ramen, tartlets, waffles, rolled wafer cookies, and cake.

Most patients introduced foods with PAL 3–5 times per week (see Figure 1 for frequency of consumption of foods with PAL by category). During follow‐up, only one reaction (1/13 = 7% of patients) was reported by a 12.1‐year‐old female with allergies to almond, cashew, pistachio, hazelnut, peanut, macadamia, and walnut. She experienced isolated itching in the mouth and throat after consuming a “tête au choco” (chocolate‐coated marshmallow puff) with PAL, which resolved without treatment.

**FIGURE 1:**
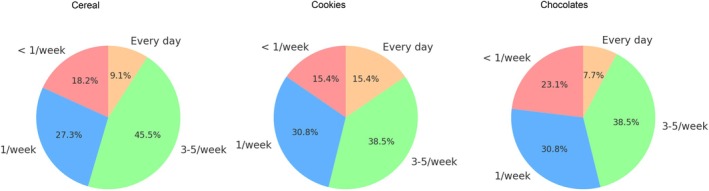
Frequency of consumption of foods with PAL after safe introduction at the clinic.

Quality of life was improved in patients after the study intervention. Median total FAQLQ scores (*n* = 13) before and after the intervention were 2.86 and 2.31 (*p* < .00001) for FAQLQ PF, 3.36 and 3.00 (*p* = .1) for FAQLQ CF, and 3.67 and 3.33 (*p* < .01) for FAQLQ TF, respectively. Specific sub‐categories of the FAQLQ including frustration with PAL were also improved (see Table 2). These findings should be interpreted as descriptive trends, as the study was not powered to demonstrate definitive clinical effectiveness.

**TABLE 2:** FAQLQ subscores improved after introduction of foods with PAL^a^.

Parents 0–12 years	
Anxiety with foods	Moderately/quite a lot to slightly/moderately
Feels different	Slightly/moderately to a little/slightly
Frightened to try non‐familiar foods	Quiet a lot/a lot to slightly/moderately
Anxious when eating with strangers	Slightly/moderately to little/slightly
Children 7–12	
Are you frustrated not be able to eat a food you would like to	Moderately/quite a lot to slightly/moderately
Are you frustrated not be able to eat treats at school	Moderately/quite a lot to slightly/moderately
Adolescents 13–17	
Are you frustrated having to restrict more foods out of home	Moderately/quite a lot to slightly
Are you frustrated having to check foods out of home	Slightly/moderately to almost not/slightly
Are you frustrated having to be careful not touching certain foods	Slightly/moderately to almost not/slightly
Are you frustrated having to be careful not touching certain foods	Slightly/moderately to almost not/slightly
Are you frustrated with PAL	Quite a lot to almost not/slightly
Are you afraid of eating a food you are allergic to	Slightly/moderately to almost not/slightly

^a^ Due to small sample size, no statistical analysis on specific questions was performed. Results highlight questions with a change of score of >1.0 (from one to the next or previous step), *n* = 13.

This pilot study suggests that, in a small and carefully selected cohort with peanut and/or tree nut allergy who exhibited only mild reactions to a 30 mg protein OFC, short‐term introduction of foods with PAL was feasible and generally well tolerated under supervised conditions. Only infrequent and mild reactions to foods with PAL occurred during both the initial challenges and the three‐month follow‐up. This could be an option for peanut/nut‐allergic patients wishing to introduce these foods without undergoing more strenuous food allergy treatments such as oral immunotherapy. Although contamination levels varied between products and batches, some hazelnut‐allergic children tolerated chocolates with detectable hazelnut, which underscores individual threshold variability and the value of personalized counseling.

Because the study was terminated early after enrolling 13 participants due to recruitment challenges, the prespecified precision targets were not achieved. Consequently, the findings are not generalizable and should be interpreted as exploratory. Other limitations include the use of a single simplified threshold dose (30 mg of protein) that may not generalize across allergens, individual threshold variability (e.g., in the presence of cofactors), and the short three‐month follow‐up. In addition, PAL practices and actual contamination levels vary widely across countries and manufacturers; therefore, the present results obtained using Swiss and Canadian food products cannot be extrapolated to other regulatory contexts. Importantly, the 30 mg protein decision‐point OFC carries a potential risk of anaphylaxis, highlighting that careful patient selection and supervised administration in low‐risk individuals are essential.

In conclusion, supervised single‐dose OFC–based risk stratification may represent a potential clinical tool to support individualized counseling regarding PAL foods in carefully selected pediatric patients, but this approach requires validation in larger, multicenter studies with diverse allergens, longer follow‐up, and international regulatory representation before broader clinical implementation can be considered.

## AUTHOR CONTRIBUTIONS

**François Graham:** Conceptualization; data curation; formal analysis; visualization; methodology; investigation; writing – original draft; writing – review and editing; validation; funding acquisition; project administration. **Mikaël Frossard:** Methodology; resources; validation; formal analysis; writing – review and editing. **Natalia Gasilova:** Formal analysis; methodology; writing – review and editing; resources; validation. **Fabienne Maréchal:** Writing – review and editing; validation; project administration; resources; methodology; data curation. **Philippe A. Eigenmann:** Conceptualization; data curation; formal analysis; visualization; writing – original draft; methodology; investigation; supervision; project administration; writing – review and editing; resources; validation; funding acquisition.

## FUNDING INFORMATION

Ulrich Müller‐Gierok Foundation, Fonds de Recherche Santé Québec, Fondation du Centre Hospitalier de l'Université de Montréal.

## CONFLICT OF INTEREST STATEMENT

None of the authors have a conflict of interest to disclose.

## Supporting information


Appendix S1.
