# Supplementary material for: Risk‐stratified introduction of precautionary allergen‐labeled foods in children with peanut and tree nut allergies
Source: Pediatr Allergy Immunol. 2026 Feb 27;37(3):e70306. doi: 10.1111/pai.70306 (PMC12948735; doi:10.1111/pai.70306)
Supplement: Supplementary file 1 — Appendix S1. [file PAI-37-e70306-s001.docx]

## **Supplementary Material**

## Appendix 1- Eligibility Criteria

Participants fulfilling all of the following inclusion criteria are eligible for the study.

Inclusion criteria

- Children 2-18 years.
- Tree nut or peanut allergy documented by:
  - Positive skin prick tests (SPTs) to peanut and/or tree nuts.
  - Positive specific IgE (sIgE) to peanut and/or tree nuts ( ≥0.35 kU/L).
  - A recent (< 1 year) positive single or double-blind food challenge, defined by a mild reaction to a dose of 30 mg of protein *, and excluding those with a moderate or severe reactions.
- Participation is subjected to written consent after appropriate information.

Exclusion criteria

- History of a moderate or severe reaction during a food challenge with a dose equal or below 30 mg of tree nut and/or peanut protein.
- Expected non-adherence to the study protocol.
- Severe or uncontrolled asthma.
- Children who are not actively avoiding foods with “may contain traces”.

*As defined by mild symptoms in Fig. 3 of PRACTALL oral food challenge guideline (1).

(1) Sampson HA, Gerth van Wijk R, Bindslev-Jensen C, Sicherer S, Teuber SS, Burks AW et al. Standardizing double-blind, placebo-controlled oral food challenges: American Academy of Allergy, Asthma & Immunology–European Academy of Allergy and Clinical Immunology PRACTALL consensus report. *J Allergy Clin Immunol* 2012;**130**:1260–1274.

## APPENDIX 2 – Analysis of foods with “May contain traces”

**A- Nut proteins extraction from food samples and sample preparation for LC-MS/MS**

1. Freezing of sample in liquid nitrogen for 5 minutes
2. Grounding of the frozen sample using a blender and/or mortar/pestle
3. Optional: defatting by addition of hexane in a 1:5 ratio (w/v), agitation for 30 min followed by filtration to recover the defatted flour and overnight drying
4. Extraction of the proteins using 20 mM Tris-HCl pH 8.2 buffer with a 1:20 ratio (w/v) for 1h at room temperature under vigorous shaking
5. Optional: ultrasound treatment for 30 min (amplitude 20 %, pulse 5 s “On”, 5 s “Off”, with cooling of the sample) (Instrument: Ultra-cell 500W, Sonics)
6. Centrifugation of the sample for 10 min and collection of the supernatant
7. Filtration through Chromafil Xtra PVDF-45/25 filter (low protein binding filter with pore size of 0.45 µm)
8. Purification of the proteins by cold acetone precipitation (overnight). Re-solubilisation of the protein pellet in a buffer suitable for enzymatic digestion (ammonium bicarbonate or Tris-HCl, pH 8)
9. Determination of total protein concentration in the sample using BCA protein assay (Thermo Fisher Scientific) with BSA as a standard
10. Denaturation of the proteins at 95°C-100°C for 10 minutes
11. Reduction of disulfide bonds by addition of 100 mM dithiothreitol and incubation at 56°C for 30 min
12. Alkylation of cysteine residues by addition of 100 mM iodoacetamide and incubation at room temperature in the dark for 30 min
13. Overnight digestion (37°C) of proteins by addition of trypsin (1 mg/mL) to a final protein:trypsin ratio of 30:1 (w/w)
14. Digestion is stopped by acidification (trifluoroacetic acid)
15. Purification of the tryptic peptides using ZipTip C_18_

**B- Identification of the extracted proteins by bottom-up proteomics**


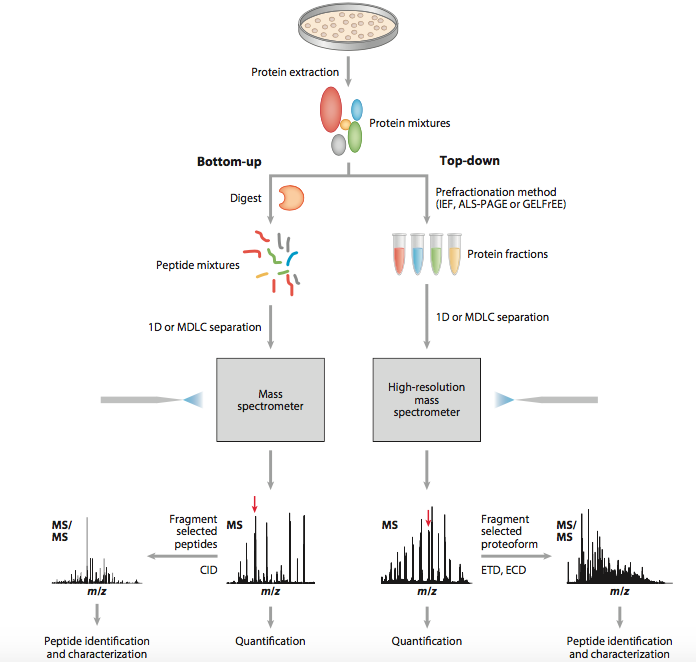


*Flowchart of bottom-up (left) and top-down (right) proteomics (Zhang et al, 2014, Annu. Rev. Anal. Chem.)*

**HPLC**: Thermo UltiMate 3000, equipped with Zorbax Extend C_18_ reverse phase column (2.1 x150 mm, Agilent Technologies) for peptides separation.

**Mass spectrometer**: Thermo Q Exactive HF Hybrid Quadrupole-Orbitrap

**C- HPLC parameters:**

Column oven temperature: 30°C

Flow rate: 0.250 mL/min

| Retention [min] | 99.9% Water, 0.1% Formic acid | 99.9% Acetonitrile, 0.1% Formic acid |
| --- | --- | --- |
| 0 | 95 | 5 |
| 3 | 95 | 5 |
| 43 | 65 | 35 |
| 44 | 20 | 80 |
| 46 | 20 | 80 |
| 47 | 95 | 5 |
| 50 | 95 | 5 |

**MS parameters:**

Resolution: 60’000

AGC Target: 3 ∙10^6^

Maximum injection time: 100 ms

Scan range: 300-1900 m/z

**MS/MS parameters:**

Microscans: 4

Resolution: 15’000

AGC Target: 1 ∙10^6^

Maximum injection time: 50 ms

TopN: 5

Isolation window: 2 m/z

**Identification parameters:**

Precursor ion mass tolerance: 10 ppm

Product ion mass tolerance: 0.04 Da

Allowed peptide modifications: deamination (N, Q), oxidation (M), phosphorylation (S, T, Y), N-term acetylation, hydroxyproline

Allowed FDR (false discovery rate, i.e. % of false positive results) 1-3%.

Minimum 2 identified peptides per protein

Database: obtained from [Uniprot](https://www.uniprot.org/)
